# Resilience of front-line facilities during COVID-19: evidence from cross-sectional rapid surveys in eight low- and middle-income countries

**DOI:** 10.1093/heapol/czad032

**Published:** 2023-05-30

**Authors:** Michael A Peters, Tashrik Ahmed, Viviane Azais, Pablo Amor Fernandez, Prativa Baral, Salomé Drouard, Rachel Neill, Kante Bachir, Poidinguem Bassounda, Queen Dube, Sabrina Flora, Edwin Montufar, Charles Nzelu, Mahamadi Tassembedo, Chea Sanford Wesseh, Bushra Alam, Jean de Dieu Rusatira, Tawab Hashemi, Alain-Desire Karibwami, Virginia Moscosco, Munirat Ogunlayi, Tania Ortiz de Zunigalo, Julie Ruel-Bergeron, Isidore Sieleunou, Peter M Hansen, Gil Shapira

**Affiliations:** The Global Financing Facility for Women, Children, and Adolescents, 1818 H St NW, Washington, DC 20433, USA; The Global Financing Facility for Women, Children, and Adolescents, 1818 H St NW, Washington, DC 20433, USA; The Global Financing Facility for Women, Children, and Adolescents, 1818 H St NW, Washington, DC 20433, USA; The Global Financing Facility for Women, Children, and Adolescents, 1818 H St NW, Washington, DC 20433, USA; The Global Financing Facility for Women, Children, and Adolescents, 1818 H St NW, Washington, DC 20433, USA; The Global Financing Facility for Women, Children, and Adolescents, 1818 H St NW, Washington, DC 20433, USA; The Global Financing Facility for Women, Children, and Adolescents, 1818 H St NW, Washington, DC 20433, USA; Ministère de la Sante de la Guinea, Blvd de Commerce, Conakry, Guinea; Ministère de la Santé Publique du Tchad, N'Djamena, Chad; Ministry of Health of Malawi, Capital Hill Circle, Lilongwe, Malawi; Government of Bangladesh Ministry of Health and Family Welfare, Abdul Gani Road, Dhaka 1000, Bangladesh; Ministerio de Salud Pública y Asistencia Social de Guatemala, Avenida 3-45, Cdad. de Guatemala, Guatemala; Federal Ministry of Health of Nigeria, Federal Secretariat Complex, Phase III, Shehu Shagari Way, Central Business District, Abuja, Nigeria; Ministère de la Santé et de l’Hygiène Publique du Burkina Faso, Ave du Burkina, Koulouba, Ouagadougou, Burkina Faso; Ministry of Health of Liberia, SKD Blvd, Monrovia, Liberia; The World Bank, 1818 H St NW, Washington, DC 20433, USA; The Global Financing Facility for Women, Children, and Adolescents, 1818 H St NW, Washington, DC 20433, USA; The Global Financing Facility for Women, Children, and Adolescents, 1818 H St NW, Washington, DC 20433, USA; The Global Financing Facility for Women, Children, and Adolescents, 1818 H St NW, Washington, DC 20433, USA; The Global Financing Facility for Women, Children, and Adolescents, 1818 H St NW, Washington, DC 20433, USA; The Global Financing Facility for Women, Children, and Adolescents, 1818 H St NW, Washington, DC 20433, USA; The Global Financing Facility for Women, Children, and Adolescents, 1818 H St NW, Washington, DC 20433, USA; The Global Financing Facility for Women, Children, and Adolescents, 1818 H St NW, Washington, DC 20433, USA; The Global Financing Facility for Women, Children, and Adolescents, 1818 H St NW, Washington, DC 20433, USA; The Global Financing Facility for Women, Children, and Adolescents, 1818 H St NW, Washington, DC 20433, USA; The World Bank, 1818 H St NW, Washington, DC 20433, USA

**Keywords:** Resilience, health facility, primary health care, COVID-19, essential health services

## Abstract

Responsive primary health-care facilities are the foundation of resilient health systems, yet little is known about facility-level processes that contribute to the continuity of essential services during a crisis. This paper describes the aspects of primary health-care facility resilience to coronavirus disease 2019 (COVID-19) in eight countries. Rapid-cycle phone surveys were conducted with health facility managers in Bangladesh, Burkina Faso, Chad, Guatemala, Guinea, Liberia, Malawi and Nigeria between August 2020 and December 2021. Responses were mapped to a validated health facility resilience framework and coded as binary variables for whether a facility demonstrated capacity in eight areas: removing barriers to accessing services, infection control, workforce, surge capacity, financing, critical infrastructure, risk communications, and medical supplies and equipment. These self-reported capacities were summarized nationally and validated with the ministries of health. The analysis of service volume data determined the outcome: maintenance of essential health services. Of primary health-care facilities, 1,453 were surveyed. Facilities maintained between 84% and 97% of the expected outpatient services, except for Bangladesh, where 69% of the expected outpatient consultations were conducted between March 2020 and December 2021. For Burkina Faso, Chad, Guatemala, Guinea and Nigeria, critical infrastructure was the largest constraint in resilience capabilities (47%, 14%, 51%, 9% and 29% of facilities demonstrated capacity, respectively). Medical supplies and equipment were the largest constraints for Liberia and Malawi (15% and 48% of facilities demonstrating capacity, respectively). In Bangladesh, the largest constraint was workforce and staffing, where 44% of facilities experienced moderate to severe challenges with human resources during the pandemic. The largest constraints in facility resilience during COVID-19 were related to health systems building blocks. These challenges likely existed before the pandemic, suggesting the need for strategic investments and reforms in core capacities of comprehensive primary health-care systems to improve resilience to future shocks.

Key messages

- Little is known about health system resilience at the facility level, despite the fact that responsive primary health care is the foundation of resilient health systems.
- This is the largest known study to describe health facility resilience experiences, capabilities and challenges during the coronavirus disease 2019 pandemic using an innovative rapid-cycle facility phone survey methodology developed and validated by eight ministries of health in Africa, Latin America and Asia.
- Findings confirm that, despite unique country challenges to providing essential health services during a health shock, the basic building blocks of primary health-care systems were critical to health system resilience and need to be continually improved.
- This study provides critical insights on how to prioritize health system strengthening investments based on the perspectives of the local actors that are responsible for the provision of primary health care.

## Background

Responsive primary health care is the foundation of resilient health systems. Health shocks, or extreme changes that can impact health facilities and broader systems, range from sudden events (e.g. disease outbreaks, conflict and natural disasters) to more protracted phenomena (e.g. epidemiological transition, migration and climate change). When shocks occur, the impact on morbidity and mortality is intensified by reductions in the utilization of essential health services.

This multiplicative ‘indirect’ effect has been described during earthquakes, hurricanes and disease outbreaks (Bartels and VanRooyen, 2012; Morita *et al.*, 2017; Sochas *et al.*, 2017; Rivera and Rolke, 2019). During the severe acute respiratory syndrome coronavirus 2 [coronavirus disease 2019 (COVID-19)] pandemic, nearly all low- and middle-income countries (LMICs) reported these disruptions, which contributed to global excess mortality (World Health Organization, 2020, 2021a, 2022). The threat of future shocks to health systems, including the resurgence of COVID-19, emphasizes the urgent need to understand the factors that enable health facilities to maintain essential services during crises. In LMIC contexts, where access to facility-based services has helped drive down maternal, newborn, and child deaths over the past few decades, health facility resilience during COVID-19 is not merely an aspiration; it is a matter of life and death.

The COVID-19 pandemic coincided with a massive increase in interest and academic research into health system resilience. This mirrors broader cycles of increased financing and attention directed towardsn more than one country. Guatemala has health systems during times of ‘panic’, followed by the periods of ‘neglect’ after health shocks are considered to be under control.

Despite the lack of consistent conceptualization of health system resilience in the literature, one definition has been frequently utilized and aligns with expert opinions on the resilience construct (Fridell *et al.*, 2020). For this paper, health system resilience is ‘The capacity of health actors, institutions, and populations to prepare for and effectively respond to crises; maintain core functions when a crisis hits; and, informed by lessons learned during the crisis, reorganise if conditions require it’ (Kruk *et al.*, 2015; 2017). As the primary point of service delivery in most health systems, the resilience of primary health-care facilities is a high priority for policymakers, researchers and, most importantly, the recipients of health services. Yet, there are major gaps in understanding the health system’s resilience at the health facility level (Li *et al.*, 2021).

A recent review mapping the interdisciplinary knowledge of health facility resilience identified two major knowledge gaps: first, that the majority of health facility resilience research comes from high-income countries, and second, that current approaches to describe facility resilience do not account for micro-level system structures such as human resources, supply chains or referral networks (Li *et al.*, 2021). As a result, it is difficult to determine the most integral components of health facility functioning and develop resilience-strengthening strategies that are effective across diverse LMIC contexts. Furthermore, most health facility resilience research has been conducted at the hospital level, which ignores the experience of lower-level primary health-care facilities in LMICs (Li *et al.*, 2021). These shortcomings are mirrored in the broader COVID-19 resilience literature, where resilience has been extensively researched at the national level. Studies have employed country-specific case studies, comparative analyses and other broader ‘lessons learned’ compendiums, providing little insight into implementation strategies to improve resilience at the local level (Chua *et al.*, 2020; Doubova *et al.*, 2021; Haldane *et al.*, 2021; World Health Organization & European Observatory on Health Systems and Policies, 2021; World Health Organization, 2021b). However, previous efforts have attempted to bridge this know-do gap by describing an implementation-oriented framework for resilient health systems (Nuzzo *et al.*, 2019; Meyer *et al.*, 2020).

Nuzzo *et al.* (2019) proposed the implementation-oriented resilience framework. This framework describes 16 themes identified through a scoping review and translates them to critical capacities for health system resilience to infectious disease outbreaks and natural hazards (Nuzzo *et al.*, 2019). Following key informant interviews and a workshop with facility-level actors, the framework was consolidated into a checklist with 10 thematic categories to support implementation (Meyer *et al.*, 2020). One of the stated purposes of the framework and subsequent checklist is to ‘flag gaps’ and motivate actionable steps to improve resilience at the health facility level (Nuzzo *et al.*, 2019; Meyer *et al.*, 2020). The implication of the checklist is that these capacities will improve preparedness, thereby improving resilience when a shock occurs.

A review of published articles citing these papers found no attempts to assess facility resilience with the framework or the checklist. Furthermore, the checklist was designed to reflect the aspects of resilience that contribute to preparedness and has not been applied to a health facility’s response to a public health shock. Thus, using an evidence-based tool that has documented face validity is an opportunity to assess the constraints in health facility resilience in LMICs, in terms of both preparedness and response to COVID-19 (Nuzzo *et al.*, 2019; Meyer *et al.*, 2020).

Understanding the components of facility-level resilience in LMICs provides insights into the constraints and strengths of COVID-19 responses at the local level and provides opportunities to strengthen health systems in the immediate and long term. This paper describes primary health-care facility resilience by applying the implementation-oriented resilience framework during COVID-19 in eight LMICs. The aspects of primary health-care facility resilience are aggregated to describe national patterns of resilience, indicating variation in response capacity and suggesting areas to improve for strengthening the response to future health shocks.

## Methods

### Study design and sampling

Since September 2020, the authors’ institute has supported the ministries of health in eight LMICs to conduct phone surveys with health facilities to understand challenges in maintaining essential health services continuity. Phone-based surveys have been increasingly used to collect data in global health research and have been validated to provide information as accurate as in-person visits for certain areas of inquiry (Pattnaik *et al.*, 2020; Khalil *et al.*, 2021). Additionally, in the context of COVID-19, phone-based methods of training and data collection reduced the risk of infection spread between enumerators and respondents. Facility phone-based surveys were conducted in Bangladesh, Burkina Faso, Chad, Guatemala, Guinea, Liberia, Malawi and Nigeria. The study was requested, reviewed and approved by a director-level official in each Ministry of Health and was exempted from human subjects research as a public health practice in every country except Burkina Faso. These eight countries represent a range of geographies, health systems and COVID-19 burdens (Table 1). A short (∼ 1-h long) telephone-based health facility survey was designed to monitor the provision of essential health services during the COVID-19 pandemic. The survey tool was developed in August 2020 in consultation with global partners, including the World Health Organization, and included modules on infrastructure, finances, supplies (including personal protective equipment), human resources, and service disruptions and adaptations (World Health Organization, 2021c). The tool was adapted based on local Ministry of Health priorities.^1^ All survey samples were randomly selected facilities from a sampling frame derived from a national registry. The result was a nationally representative sample of public health facilities (i.e. public hospitals, health centres and lower-level or community clinics) in each country, and minor variations in sampling approaches are described in Supplementary Appendix 1. Facilities were considered for inclusion if they were included in the national registry of facilities provided by the Ministry of Health.

**Table 1.** Characteristics of studied countries

	**Health system description**					**COVID-19 burden**		
**Country**	**Universal Health Coverage service index (2019—out of 100)** ^a^	**Incidence (%) of catastrophic health expenditure (at 10% of household spending level)** ^a^	**Health workforce distribution (physicians per 1000 people)** ^a^	**Per capita government spending on health (PPP, current international $)** ^a^	**Role of the private sector** (**percentage of diarrhoea care delivered by the private sector)**^b^^,^^c^	**COVID-19 deaths per million people** **(as of December 2021**)^d^	**COVID-19 peak date (as of December 2021**)^d^	**COVID-19 peak number of new daily cases (7-day average, as of December 2021**)^d^
Bangladesh	51	24.7	0.64	18.6	0.87	175	Aug-21	14 477
Burkina Faso	43	3.1	0.09	47.5	0.04	18	Jan-22	286
Chad	28	6.3	0.05	13.4	0.03	191	Dec-21	85
Guatemala	57	1.4	0.35	173.9	0.35	983	Aug-21	3983
Guinea	37	7.0	0.08	18.0	0.15	33	Jan-22	298
Liberia	42	N/A	0.04	25.9	0.33	57	Jul-21	161
Malawi	48	4.2	0.04	34.5	0.17	135	Jan-21	994
Nigeria	44	15.1	0.38	34.6	0.53	15	Dec-21	2011

a Source: World Bank World Development Indicators.

b Source: Grepin KA (2016).

c Source: Most recent year for which data are available: 2011 Bangladesh; 2010 Burkina Faso; 2004 Chad; 1998 Guatemala; 2012 Guinea; 2013 Liberia; 2010 Malawi and 2013 Nigeria.

d Source: JHU CSSE COVID-19 Data. Accessed 28 December 2021.

### Data collection

**Figure 1.**
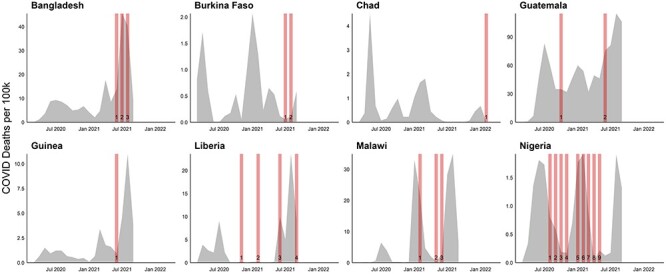
The timing of survey rounds and the national COVID-19 burden

Multiple attempts were made to reach each facility, and interview times were scheduled in advance to minimize the burden on the respondents. In case of non-response, a replacement facility of the same facility level was randomly selected from the same administrative unit. Local enumerators were hired, trained in the survey tool and the administration of phone-based surveys and then piloted the tool in each study context before survey implementation. Survey respondents generally included the facility officer incharges, but in some cases, other respondents, like facility administrators, were better suited to answer modules within the survey. Survey participation was voluntary and verbal consent was received from all respondents. The survey was administered roughly quarterly in the eight countries between August 2020 and December 2021 depending on the frequency of Ministry of Health requests for data (Figure 1).

Shaded plots represent the number of COVID-19 deaths per 100 000 people over time. The vertical bars indicate time periods for the survey round (indicated by the black numeral).

The phone survey results were mapped ex post to a framework describing health system resilience at the facility level developed and validated by Nuzzo *et al.* (2019) (Table 2). The framework suggests 10 domains of resilience capacities, with proposed measurement indicators. An additional capacity was included to assess the availability of essential medicines and supplies. This change was based on feedback from respondents and Ministry of Health stakeholders, who indicated that supply shortages were a major barrier to maintaining essential health services during COVID-19. The originally designed survey tool did not collect information on the leadership and command structures of facilities or the level of communication, collaboration, coordination and partnerships at facilities, so these two domains were not included in the final analysis. Resilience domains were mostly scored by a binary variable, where ‘1’ means that a facility demonstrates capacity based on responses to the phone survey. Where multiple survey rounds were conducted in a country, responses from the first round where a question was asked were used for the analysis.

**Table 2.** Resilience domain definition and indicator

Resilience domain	Definition	Indicator
Core health system capacities and capabilities	The capacities and capabilities needed to maintain core functioning during a public health emergency, such as access to maternal–child care.	Percent of the volume of outpatient services utilized between March 2020 and December 2021 compared to the expected volume (based on pre-pandemic seasonality and trends between January 2018 and February 2020)
Barriers to accessing health services	Barriers that exist might prevent individuals from accessing care routinely and during public health emergencies.	Percent of facilities that increased the frequency of any outreach activities (e.g. immunization, malaria prevention, NTDs, NCDs, community clinics or home visits) or made adaptations to expand access to services (e.g. providing care in a single visit for multiple morbidities, providing home-based care, shifting clinical encounters to digital platforms and using novel dispensing approaches for medicines) during the pandemic
Workforce	The health workforce and associated capacities and capabilities, including training and support, needed to respond to a public health emergency.	Percent of facilities that did not report moderate to severe challenges with human resources during the pandemic
Infection control	The infection control processes and procedures needed to prevent the spread of disease and screen and treat patients within facilities.	Percent of facilities that had masks (N95 or surgical masks) and gloves available and handwashing stations available during the first survey round
Surge capacity	Policies, practices and systems necessary to accommodate a surge of patients during a public health emergency.	Percent of facilities that recruited new staff, volunteers or seconded staff during the pandemic or were able to refer a COVID-19 case to another location
Financing	The presence of adequate resources to both maintain routine services and respond to public health crises.	Percent of facilities that did not report moderate to severe challenges with financing during the pandemic
Critical infrastructure and transportation	The infrastructure (e.g. water and sanitation) and transportation must be in place to ensure continued functioning during an emergency.	Percent of facilities that had continuous access to water and electricity the week before the survey and had access to safe and isolated transportation for patients with COVID-19
Risk communication	Policies and practices for communicating and engaging with the public about public health emergencies.	Percent of facilities that discussed COVID-19 vaccination with patients and the community
Medical supplies and equipment	A resilient health system has access to medical supplies and equipment, including personal protective equipment, antivirals and ventilators, during a crisis.	Percent of facilities that did not report moderate to severe challenges with supplies during the pandemic
Communication, collaboration, coordination and partnerships	Relationships that exist with response partners before and during a public health emergency.	N/A
Leadership and command structure	The leadership, command and incident management structures and policies needed to respond to a public health crisis.	N/A

NCDs, non-communicable diseases; NTDs, neglected tropical diseases.

### Analysis approach

One indicator from the framework (core health system capacities and capabilities) was considered an outcome measure of resilience, whereas the other domains were considered as inputs to resilience. The outcome indicator demonstrates a facility’s ability to maintain core functions when a crisis hits and is aligned with previous definitions of resilience (Kruk *et al.*, 2017). The measure for this indicator was calculated from a previously reported analysis of routine health facility data also conducted by the study team, where the predicted volume of outpatient consultations is estimated based on historical monthly routine health service volume data. The methods for this analysis have been described elsewhere (Shapira *et al.*, 2021; Ahmed *et al.*, 2022). Briefly, the observed volume of outpatient consultations at the facility level is compared with this predicted value to estimate the percent shortfall between March 2020 and December 2021. The score was a continuous number between 0 and 1, where 0 represents 0% of the expected services provided and 1 represents 100% or greater of the expected volume of services provided between March 2020 and December 2021. The national score for this domain is the simple average of facility shortfalls. For Chad, the core health system capacities and capabilities domain was not included due to a lack of sufficient data. Missing data were excluded from all analyses.

Facilities across different health systems were classified into lower-level or community clinics, health centres and hospitals (Supplementary Appendix 2). The analysis included health centers and lower-level facilities (e.g., community clinics): results for hospitals are presented in the Supplementary appendix due to differences in survey design and functionality of hospitals vs primary care clinics. The average score within a country represents the percent of primary care facilities demonstrating capacity in a given resilience domain. Resilience scores were presented by domain across countries. An overall resilience score was calculated by taking the arithmetic mean of all available domains. For easy comparison, resilience scores were visualized in radar charts across countries and geographies. All analyses were completed in Stata version 17.0, and visualization was completed in Tableau version 2020.3.2.

A series of sensitivity tests were conducted to assess the stability of results based on (1) alternative definitions of resilience domains and (2) different survey time points. For the first check, resilience scores were compared with scores calculated using alternative (second-best) indicator definitions, which were available for five of the nine resilience domains (see Supplementary Appendix 6 for details). To assess the consistency of resilience scores across the original and alternative indicator definitions, a simple percentage agreement between each pair of the original and alternative indicators was computed. The percentage agreement corresponds to the share of facilities in each country that, for a given resilience domain, had an identical score in the original and alternative resilience measures. The second sensitivity check tested the stability of selected resilience indicators over time to ensure that resilience patterns were robust to changes in country-specific contexts (e.g. varying levels of COVID-19 burden over time). Resilience scores in the first round of surveys in each country were compared to the average scores across all subsequent survey rounds with available data.

## Results

### Final sample

The final sample included 1453 facilities across the eight countries (Table 3). Most sampled facilities were from rural locations, and over half of the facilities were health centres rather than community-level facilities. The response rate ranged from 70% in Nigeria to 100% in Burkina Faso. Individual survey questions were collated, and results were stratified by country and round (Supplementary Appendix 3). The most frequently available resilience domain was barriers to access, which was measured in 1437 facilities (99%) across the eight countries. Information on risk communication was not collected in two countries, but no other domain was missing in more than one country. Guatemala has the most gaps in resilience domains (five out of nine available) (Supplementary Appendix 4).

**Table 3.** Phone survey sample characteristics

Country	Number of PHC facilities included in the survey	Sample frame (number of PHC facilities reporting in HMIS)	Percent of the sample comprised rural facilities	Percent of the sample comprised health centres	Percent of the sample comprised lower-level facilities	Percent of the response rate across rounds
Bangladesh	295	14 639	62.4	65.9	34.1	95–98
Burkina Faso	159	3028	90.6	100.0	0	99–100
Chad	117	NA	77.8	100.0	0	79
Guatemala	245	2013	83.1	15.0	85.0	93–97
Guinea	162	427	75.6	96.6	3.4	92–100
Liberia	111	610	85.3	11.3	88.8	95–98
Malawi	144	699	95.0	100.0	0	92–100
Nigeria	220	31 531	76.2	55.6	44.4	70–89
Total	1453	NA	78.1	61.4	38.6	

HMIS: health managemetn information system; PHC: primary health care.

Country-specific classifications of health centres and lower-level facilities can be found in Supplementary Appendix 3.

### Resilience capacities

Core health system capacity, or the ability to maintain essential health services during the pandemic, was the primary outcome measure of facility-level resilience. Countries delivered an average of 88% of the expected outpatient service volume between March 2020 and December 2021 compared to predicted volumes based on time trends and seasonality (Table 4). Facilities in Bangladesh had a notable shortfall in service volume, delivering 69% of the expected outpatient services during the first 20 months of the pandemic. Guatemala (84%) and Nigeria (86%) also experienced severe shortfalls in the expected service volume at the primary care level. Liberia (97%), Malawi (96%), Burkina Faso (95%) and Guinea (93%) provided >90% of the expected service volume, suggesting that this set of countries was generally able to maintain essential health services during the pandemic.

**Table 4.** Health facility resilience scores

	Outcome	Resilience domains								
Country	Core health system capacities (%)	Barriers to accessing services (%)	Infection control (%)	Workforce (%)	Surge capacity (%)	Financing (%)	Critical infrastructure (%)	Risk communications (%)	Medical supplies and equipment (%)	Average across the domains (%)
Bangladesh	69	96	59	44	N/A	78	N/A	87	66	72
Burkina Faso	95	63	86	65	82	50	47	86	57	67
Chad	N/A	14	77	38	93	37	14	90	43	51
Guatemala	84	96	70	N/A	89	N/A	51	N/A	N/A	77
Guinea	93	72	43	81	67	73	9	N/A	73	60
Liberia	97	94	75	51	99	48	54	94	15	66
Malawi	96	78	97	67	93	81	77	83	48	78
Nigeria	86	72	86	52	60	35	29	49	44	54
Total	88	77	75	54	69	58	41	80	53	63

The data source for core health system capacities is the HMIS service volume. The data source for all other resilience domains is the facility-level response to the rapid-cycle phone survey.

**Figure 2.**
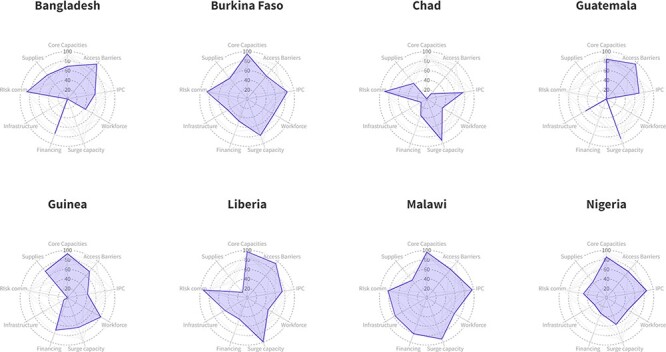
Presentation of resilience capacities by country

Despite variation in the magnitude of service disruption, facilities across countries demonstrated somewhat similar patterns in resilience domains, though major constraints differed across countries (Figure 2). Across the resilience domains, facilities in Malawi demonstrated the highest overall resilience score (0.78), and facilities in Chad had the lowest score (0.51) (Table 4). The most consistent overall strength across countries was risk communication, specifically the ability of facility health workers to communicate the risks and benefits of COVID-19 vaccination. Across all facilities, critical infrastructure was the largest overall constraint to resilience: 41% of surveyed facilities had continuous access to water, electricity, and safe and isolated transportation for patients with COVID-19. In Burkina Faso, Chad (tied), Guatemala, Guinea and Nigeria, critical infrastructure was the largest reported barrier to resilience. As few as 14% of facilities in Chad and 9% of facilities in Guinea met these criteria, with access to transportation being the weak point in critical infrastructure for facilities in both countries. Supplies (53% of facilities demonstrating capacity), workforce (54% of facilities demonstrating capacity) and financing (58% of facilities demonstrating capacity) also proved to be relatively weak points across countries and domains. Challenges with medical supplies and equipment availability were the largest resilience constraint for facilities in Liberia and Malawi (15% of facilities not reporting supply challenges in Liberia and 48% of facilities not reporting supply challenges in Malawi). In Bangladesh, the health workforce was the largest constraint (44% of facilities reporting challenges with human resources during the pandemic). In addition to challenges with critical infrastructure, facilities in Chad also had major shortcomings in their ability to reduce barriers to access services (14% of facilities made such adjustments).

Scores in resilience capacities are charted from 0 to 1 and presented on the following spider chart. Missing data are represented by a lack of red dot on the respective capacity axis.

### Sensitivity tests

The stability of indicators was found to be relatively weak based on alternative domain definitions, but results were consistent over time. The overall percent agreement between the selected and alternative indicator definitions ranged from 46% for the ‘infection prevention and control’ domain to 69% for the barriers to access domain (Supplementary Appendix Tables 5.1 and 5.2). Resilience scores in the first round of surveys in each country were compared to the average scores across all subsequent survey rounds with available data. Despite some variation in resilience scores over time within countries, resilience patterns were overall stable, with the rankings of resilience scores being significantly correlated between the first and subsequent rounds of surveys in most countries (Supplementary Appendix Table 5.3).

## Discussion

Findings from this multi-country research effort suggest that the largest constraints to facility resilience during COVID-19 were related to health system building blocks—critical infrastructure, medical supplies and equipment, health workforce and financing—and that health facilities maintained essential health service volumes with varying levels of success. Although health facility resilience in response to COVID-19 varied between countries, cross-country patterns emerged from our data.

Compared to facility-level constraints in basic health system inputs, separate analyses of this same survey data strengthens the assertion that facilities benefited from intense COVID-19-related efforts to improve infection control and vaccine-focused risk communications (Baral, 2023; Drouard, 2023). Facilities were generally able to change services to remove barriers to access during the pandemic, suggesting a relatively strong ability to be adaptive to external surroundings. However, despite these adaptive capacities, facilities in all eight study settings experienced cumulative disruptions to health service volumes, as demonstrated by the core health system capacities and capabilities domain. About half of all facilities reported a lack of capacity in critical infrastructure, medical supplies and equipment, workforce and financing during the COVID-19 pandemic. While certain health workers and facilities adapted by finding new partners to provide essential medicines and even bringing their own supplies to facilities, on aggregate, these existing weaknesses may have been exacerbated during COVID-19. Core health system input limitations may have then hamstrung facilities’ overall resilience abilities to adapt and respond during the pandemic (Kruk *et al.*, 2017).

Resilient health systems should be integrated, adaptive and diverse, leveraging foundational universal health coverage and primary health-care capacities to support the delivery of quality services (Kruk *et al.*, 2017). Several authors have built upon Kruk’s original framework to describe how governance at multiple levels and domains of resilience intersect to produce resilient health systems (Lebel *et al.*, 2006; Blanchet *et al.*, 2017; Grimm *et al.*, 2021). Our findings emphasize how broader primary health-care weaknesses may have undermined the ability of individual health facilities to be resilient during the COVID-19 pandemic. Other facility-based assessments have found similar gaps in staffing, infrastructure, and medicines and commodities during routine health system operations, suggesting that these primary health-care weaknesses predated the pandemic’s onset (O’Neill *et al.*, 2013). In the COVID-19 context, however, these systemic gaps were perceived by health facility managers to constrain their ability to be resilient. Our COVID-19-specific findings therefore align with existing research that has emphasized both the adaptive and foundational resilience capacities of front-line health workers and the health system elements that limit their support (Witter *et al.*, 2017; Kagwanja *et al.*, 2020; Grimm *et al.*, 2021). These basic health system inputs cannot be quickly improved during a period of response to a crisis but rather require sustained investments (Grimm *et al.*, 2021; Neill *et al.*, 2022).

Other scholars have emphasized the need for a complex adaptive systems approach to understanding resilience, focused on ‘creating the conditions that enable system’s effectiveness’ (Barasa *et al.*, 2017). Rather than targeting improvement in a specific domain, this study suggests the importance of broader health system strengthening approaches. This is evidenced by the often–complex relationship between resilience with the causes of service disruption. In some countries, the upstream causes of service disruptions were easily identified. Lack of medical supplies and equipment was the largest resilience constraint in Liberia, and facility managers also reported that the lack of essential drugs was the primary reason for service disruptions. In this case, the priority area for intervention is clear, yet in other cases, linkages are less straightforward. Bangladesh and Guatemala scored the highest on their ability to remove barriers to accessing services during the pandemic, yet these were the countries that experienced the largest disruptions to essential health services. Systems-level interventions are then needed to generate demand and ensure that health services are acceptable to patients to address the disruptions in service volume. These findings complicate an instrumentalist view of resilience and reinforce the need for systems thinking, an argument that has been made elsewhere (Barasa *et al.*, 2017; Khan *et al.*, 2018; Sturmberg, 2018; Saulnier *et al.*, 2021).

This study presents methodological and conceptual advancements to existing frameworks for measuring resilience at the facility level (Kruk *et al.*, 2015; Nuzzo *et al.*, 2019; Meyer *et al.*, 2020). First, the core health system capacities domain is considered an ‘outcome’ measure of resilience compared to other measures that contribute to overall resilience. Our interpretation of domains within the original resilience framework represents resilience as both a process and an outcome, reflecting the complex and dynamic nature of the concept of resilience. Since maintaining essential health service volumes is an outcome measure of health system resilience, this study supports the impetus for routine monitoring of service volume through health information systems beyond the COVID-19 pandemic. Second, based on feedback from the ministries of health, the availability of medical supplies and equipment was reintroduced to the framework as a key domain of resilience. Third, we found that domains of facility resilience are highly sensitive to definition changes. We considered indicators that reflected both preparedness and response to COVID-19 and found that changes in indicator definition caused variations in results. In the broader literature, more work needs to be done to define the measurable aspects of facility resilience that contribute to improved performance during a crisis, including whether resilience definitions should change based on the kind of shock experienced. In future work, the relative weights and importance of resilience domains should be considered and may be country-specific based on the operating context and priorities of the health system. Finally, other studies have indicated the importance of leadership, coordination and other ‘soft’ contributors to health system resilience, and future work should develop methodologies for capturing these competencies at the health facility and local levels (Barasa *et al.*, 2018). These advances should be considered within the limitations of the study.

In addition to the previously described effect of the definitions of resilience on findings, several constraints should be considered. The timing of surveys, different burdens of COVID-19 within and across countries and various levels of government stringency measures mean that facilities were exposed to varying levels of health shock during the study period (Shapira *et al.*, 2021).^.^Since most resilience domain questions attempted to determine shocks and capacities since the beginning of the pandemic, the period of the highest shock (the second quarter of 2020) is incorporated for all facilities, regardless of survey round timing (Shapira *et al.*, 2021). Furthermore, while most of the countries in the study had similar stringency indices during the study period, Bangladesh’s notably more stringent control measures may contribute to the higher levels of service disruption observed in the country (Mathieu *et al*., 2020). Although we may expect certain domains to vary substantially over time as health systems adapt to challenges, sensitivity checks demonstrate that certain signals identified in the survey are stable, regardless of differences in COVID-19 burden over time (Supplementary Appendix Table 6.3). Another limitation of this study is the lack of complete data. The survey tool omitted two resilience domains (leadership and command structure; communication, collaboration, coordination and partnerships). Therefore, we cannot indicate the capacity level of these essential ‘software’ health system capabilities during the pandemic. We also cannot speak to the characteristics of resilience from the community perspective, which would help to supplement the findings of this supply-side study (Alonge *et al.*, 2019; Bhandari and Alonge, 2020). Additionally, the slight variation in survey design and implementation means that some countries have missing data for certain resilience domains. The primary purpose of the research activity was to be responsive to government needs and minimize the burden on facility respondents, so these gaps in data availability, while unfortunate for this study, reflect the priorities of the study partners. Finally, the self-reported nature of the phone survey may result in some bias compared to directly observed results. Desirability bias and information bias may potentially positively bias results; however, results were given face validity through conversations with Ministry of Health officials. Future work should validate responses obtained from rapid-cycle phone surveys relative to in-person assessments.

By taking a bottom-up approach and enlisting the experiences of front-line primary health-care facility workers across eight countries, this study contributes to the burgeoning field of health system resilience. In so doing, the voices of local health system actors, a traditionally marginalized group in the field of health systems resilience, are amplified and emphasized (Chopra and Kasper, 2021). This study also illustrates the critical importance of conducting resilience research across the levels of the health system. While our study took a facility-centric approach, the findings emphasized the interdependencies between health facility adaptations and higher-order health system capacities. Implementation research studies, such as positive deviance analyses, can describe the relationship between resilience domains and improved performance during the pandemic. These studies can help further elucidate the relationships between the resilience domains and the interventions and actions across the levels of the health system, which improve the resilience during shocks.

Findings also emphasize the value of the rapid-cycle phone surveys utilized in this study to continuously monitor service volume levels and causes for challenges beyond response to specific shocks. This is necessary to monitor iterative progress to strengthen health systems at the local level, where services are delivered. This study suggests that such efforts would contribute to filling the largest gaps in the health system’s resilience—the routine capacity of primary health-care systems to deliver quality services—and provide critical insights into policymakers on how to prioritize health system strengthening investments.

## Conclusion

This study provides evidence that the gaps in resilience among primary health-care facilities in LMICs were not related to failures to make the necessary COVID-19 adaptations but rather originated from historical health system weaknesses that were exacerbated by the pandemic. Within countries, efforts should be focused on regularly improving these core systems, as these health system investments will also fill the largest gaps in terms of facility resilience to health shocks. Only when routine commitment to strengthening health systems matches efforts during times of shock can the cycles of panic and neglect be broken.

## Supplementary Material

czad032_Supp

## Data Availability

The data underlying this article are available in the article and in its online supplementary material.

## Footnotes

1. For example, one country that observed a high number of staff absenteeism over the course of the pandemic added questions about reasons for vacant posts.

## Reflexivity statement

This research was designed to rapidly provide data and context to answer specific policy questions about how health facilities were responding to the COVID-19 pandemic and the challenges they faced in providing services in eight LMICs. The data collection tool gave voice to local health facility managers, by allowing them to directly report their challenges to the ministries of health, who used findings to guide COVID-19 response measures. The GFF utilizes a country-led approach to improve outcomes for vulnerable populations and supported LMICs to monitor and quantify the effect of the COVID-19 pandemic on essential health services for women, children and adolescents. Ministry of Health officials in eight LMICs (K.B. from Guinea, P.B. in Chad, Q.D. in Malawi, S.F. in Bangladesh, E.M. in Guatemala, C.N. in Nigeria, M.T. in Burkina Faso and C.S.W. in Liberia) requested GFF support and identified local priorities for investigation, led the adaptation of a global WHO–standardized tool to local contexts, guided the implementation of the survey (i.e. the sampling process and frequency) and validated the findings in real time. Researchers from the GFF’s central team (M.A.P., T.A., V.A., P.A.F., P.B., S.D., R.N., T.H., P.M.H. and G.S.) provided analytical and technical support, while researchers from GFF’s country teams (B.A., J.d.D.R., A.-D.K., V.M., M.O., T.O.d.Z., J.R.B. and I.S.) facilitated local adaptation and considerations. Even though many of the GFF and World Bank authors have diverse cultural heritages, originate from and currently live and work in LMICs, researchers from organizations based in high-income countries are over-represented on the list of authors of this article. The first two authors (M.A.P. and T.A.) have only recently graduated from PhD programs and can be considered early career researchers. Several other early career researchers (V.A., P.A.F., P.B., S.D. and R.N.) were included in the authorship team. They contributed to the implementation of surveys and the harmonization and synthesis of results. We acknowledge that most of them are based in high-income countries. Thirteen authors are female (V.A., P.B., S.D., R.N., K.B., P.B., Q.D., S.F., B.A., V.M., M.O., T.O.d.Z. and J.R.B.) and thirteen authors are male (M.A.P., T.A., P.A.F., P.B., E.M., C.N., M.T., C.S.W., J.d.D.R., T.H., A.-D.K., I.S., P.M.H. and G.S.).
