# Supplementary material for: Resilience of front-line facilities during COVID-19: evidence from cross-sectional rapid surveys in eight low- and middle-income countries
Source: Health Policy Plan. 2023 May 30;38(7):789–98. doi: 10.1093/heapol/czad032 (PMC11318646; doi:10.1093/heapol/czad032)
Supplement: czad032_Supp [file czad032_supp.zip › suppl_data/Appendix Tables.docx]

**Appendix 1: Sampling strategy for included countries**

| **Country** | **Sampling strategy** | **Overall N** |
| --- | --- | --- |
| **Bangladesh** | 20 districts were randomly sampled, with probability proportional to size, from which 100 Upazilas were randomly selected, at 5 per district. Three facilities per Upazila were subsequently selected (one Upazila health complex, and a random selection of a union-level facility and a community clinic). | 295 |
| **Burkina Faso** | Random sample drawn of public health centers and health posts. Sample stratified by province and facility type. | 159 |
| **Chad** | Random sample drawn of public health centers and health posts. Sample stratified by province and facility type. | 117 |
| **Guatemala** | Random sample drawn of public health centers and health posts. Sample stratified by province and facility type. | 245 |
| **Guinea** | Random sample drawn of public health centers and health posts. Sample stratified by province and facility type. | 162 |
| **Liberia** | Random sample drawn of public health centers and clinics. Sample stratified by county and facility type. | 111 |
| **Malawi** | Random sample drawn of public health centers. Sample stratified by district, facility type, and ownership (government / CHAM). CHAM facilities are oversampled. Health posts, clinics, and dispensaries are folded into a community level stratum, and were not included in the selection. | 144 |
| **Nigeria** | Random sample drawn of private and public health centers, clinics, and maternity homes. Sample stratified by state and facility type. The regions of Lagos, FCT and Rivers were oversampled. | 220 |

**Appendix 2: Classification of health facilities, by country**

|  | **Facility location** | | | **Facility ownership** | | | **Facility type** | |
| --- | --- | --- | --- | --- | --- | --- | --- | --- |
|  | *Initial* | *Classified as* | *Initial* | | *Classified as* | *Initial* | | *Classified as* |
| Bangladesh | Urban | Urban | All public | | Public | Upazila health complex | | Health center |
|  | Peri/ex-urban | Urban |  | |  | Union health and family welfare center | | Health center |
|  | Rural | Rural |  | |  | Community clinic | | Lower-level or community clinic |
| Burkina Faso | Urban | Urban | All public | | Public | Medical center | | Health center |
|  | Peri/ex-urban | Urban |  | |  | CSPS | | Health center |
|  | Rural | Rural |  | |  |  | |  |
| Chad | Urban | Urban | Public | | Public | Hospital | | Hospital |
|  | Peri/ex-urban | Urban | Private | | Private | Health center | | Health center |
|  | Rural | Rural | Confessional | | Private | Health post | | Lower-level or community clinic |
| Guatemala | Urban | Urban | All public | | Public | District hospital | | Hospital |
|  | Peri/ex-urban | Urban |  | |  | Health center | | Health center |
|  | Rural | Rural |  | |  | Maternal and Child Comprehensive Care Center (CAIMI) | | Health center |
|  |  |  |  | |  | Permanent Care Center (CAP) | | Health center |
|  |  |  |  | |  | Outpatient Care Center (CENAPA) | | Health center |
|  |  |  |  | |  | Strengthened Health Post (PSF) | | Lower-level or community clinic |
|  |  |  |  | |  | Health post | | Lower-level or community clinic |
| Guinea | Urban | Urban | All public | | Public | Regional/prefectural hospital | | Hospital |
|  | Rural | Rural |  | |  | Health Center/CSA | | Health center |
|  |  |  |  | |  | Communal medical center (CMC)/Health post | | Lower-level or community clinic |
| Liberia | Urban | Urban | All public | | Public | Hospital | | Hospital |
|  | Rural | Rural |  | |  | Health center | | Health center |
|  |  |  |  | |  | Clinic | | Lower-level or community clinic |
| Malawi | Urban | Urban | Government | | Public | Hospital/district hospital | | Hospital |
|  | Peri/Ex-urban | Urban | Private for profit | | Private | Health center | | Health center |
|  | Rural | Rural | Private not for profit (e.g., CHAM, IHAM, BLM, company) | | Private | Community/rural hospital | | Health center |
| Nigeria | Urban | Urban | Public Primary Health Care | | Public | Hospital | | Hospital |
|  | Peri/Ex-urban | Urban | Secondary Health Care | | Public | Health center | | Health center |
|  | Rural | Rural | Private Primary Health Care | | Private | Health post | | Lower-level or community clinic |
|  |  |  |  | |  | Maternity homes/clinics | | Lower-level or community clinic |
|  |  |  |  | |  | Health care/primary health care | | Health center |
|  |  |  |  | |  | Hospital and maternity | | Health center |
|  |  |  |  | |  | Church, school, dispensary, laboratory, nursing home | | Removed |

**Appendix 3: Survey results master table**

**[External Excel Document – Sheet 2]**

**Appendix 4: Count of facility responses by country**

| **Country** | **Barriers to access** | **Infection control** | **Workforce** | **Surge capacity** | **Financing** | **Critical infrastructure** | **Risk communications** | **Supplies** |
| --- | --- | --- | --- | --- | --- | --- | --- | --- |
| Bangladesh | 295 | 239 | 295 | 0 | 295 | 0 | 295 | 295 |
| Burkina Faso | 159 | 145 | 133 | 159 | 133 | 159 | 159 | 133 |
| Chad | 117 | 86 | 117 | 90 | 117 | 116 | 115 | 117 |
| Guatemala | 245 | 224 | 0 | 229 | 0 | 245 | 0 | 0 |
| Guinea | 151 | 72 | 153 | 150 | 153 | 150 | 0 | 153 |
| Liberia | 111 | 110 | 95 | 102 | 95 | 111 | 102 | 95 |
| Malawi | 144 | 143 | 42 | 120 | 42 | 144 | 120 | 42 |
| Nigeria | 215 | 161 | 216 | 180 | 216 | 218 | 191 | 216 |
| **Total** | 1437 | 1180 | 1051 | 1323 | 1051 | 1143 | 982 | 1051 |

**Appendix 5: Sensitivity checks**

**Appendix 5.1. Alternative (second-best) indicator definitions for 5 resilience domains**

| **Domain** | **Second-best Indicator** |
| --- | --- |
| **Barriers to accessing health services** | Percent of facilities that either (i) registered patients who have missed appointments or (ii) planned for catch-up of missed appointments for high-risk patients or (iii) planned for catch-up community outreach activities |
| **Infection Prevention and Control** | Percent of facilities that implemented all the following 3 IPC protocols: (i) screening of patients and staff at a dedicated entrance; (ii) environment cleaning and disinfection using SOPs; and (iii) clear separation of COVID-19 areas |
| **Workforce** | Percent of facilities that (i) did not experience an increase in staff absences during the pandemic and that (ii) provided mental health and psychological resources or training on emergency conditions management to their staff |
| **Medical supplies and Equipment** | Percent of facilities that did not experience service disruptions due to a supply stockout in the past month |
| **Financing** | Percent of facilities that reported their level of funding was sufficient for current needs |

**Note:** Alternative indicator definitions could only be identified for 5 of the 9 resilience domains.

**Appendix 5.2. Percentage agreement of resilience scores between original and alternative domain definitions, by country**

| Country | Resilience definition | Barriers to access | Financing | Health Workforce | IPC | Supplies | **Country average** |
| --- | --- | --- | --- | --- | --- | --- | --- |
| Bangladesh | Original definition | 96% | 78% | 44% | 59% | 66% | 69% |
|  | Alternative | 34% | - | 56% | 36% | 80% | 52% |
|  | *% Match* | 37% | - | 45% | 61% | 67% | 53% |
| Burkina Faso | Original definition | 63% | 50% | 65% | 86% | 57% | 64% |
|  | Alternative | 74% | 6% | 68% | 21% | 92% | 52% |
|  | *% Match* | 67% | 55% | 53% | 33% | 56% | 53% |
| Chad | Original definition | 14% | 37% | 38% | 77% | 43% | 42% |
|  | Alternative | 69% | 11% | 34% | 2% | 81% | 39% |
|  | *% Match* | 42% | 64% | 66% | 26% | 48% | 49% |
| Guatemala | Original definition | 96% | - | - | 70% | - | 83% |
|  | Alternative | 97% | 26% | 64% | 48% | 85% | 64% |
|  | *% Match* | 95% | - | - | 54% | - | 75% |
| Guinea | Original definition | 72% | 73% | 81% | 43% | 73% | 68% |
|  | Alternative | 96% | - | 28% | 5% | 91% | 55% |
|  | *% Match* | 74% | - | 31% | 58% | 73% | 59% |
| Liberia | Original definition | 94% | 48% | 51% | 75% | 15% | 57% |
|  | Alternative | 96% | 3% | 78% | 54% | 14% | 49% |
|  | *% Match* | 90% | 54% | 58% | 54% | 77% | 67% |
| Malawi | Original definition | 78% | 81% | 67% | 97% | 48% | 74% |
|  | Alternative | 88% | - | 71% | 62% | 55% | 69% |
|  | *% Match* | 70% | - | 63% | 59% | 43% | 59% |
| Nigeria | Original definition | 72% | 35% | 52% | 86% | 44% | 54% |
|  | Alternative | 72% | 11% | 49% | 7% | 70% | 42% |
|  | *% Match* | 77% | 62% | 55% | 20% | 49% | 56% |
| **Domain Average Score** | Original definition | 73% | 43% | 57% | 74% | 49% | 59% |
|  | Alternative | 78% | 8% | 55% | 29% | 69% | 48% |
|  | ***% Match*** | 69% | 59% | 53% | 46% | 59% | **57%** |

**Note:** The percentage agreement corresponds to the share of facilities in a country that had identical or “matching” scores for each pair of alternative resilience domain definitions (i.e., taking either the value 0 or 1 in both alternative domain definitions). Scores that are available for one type of measure only (I.e., either selected or alternative) were excluded from average calculations.

**Appendix 5.3. Stability of resilience scores between the 1st available round of survey and the average over subsequent rounds, by country**

| Country | Resilience definition | Barriers to access | IPC | Health Workforce | Supplies | Financing | Infrastructure | Risk communication | **Spearman’s Rho (rank correlation coefficient)** |
| --- | --- | --- | --- | --- | --- | --- | --- | --- | --- |
| Bangladesh | First available round | 96% | 59% | 44% | 66% | 78% | - | 87% | **0.9** |
|  | Average over subsequent rounds |  | 69% | 57% | 80% | 76% | - | 90% |  |
| Burkina Faso | First available round | 63% | 86% | 65% | 57% | 50% | 47% | 86% | **0.7** |
|  | Average over subsequent rounds | 49% | 82% | 68% | 71% | 68% | 54% | 91% |  |
| Guatemala | First available round | 96% | 70% | - | - | - | 51% | - | **-**  **-** |
|  | Average over subsequent rounds | 89% | - | - | - | - | 38% | - |  |
| Guinea | First available round | 72% | 43% | 81% | 73% | 73% | 9% | - | **-** |
|  | Average over subsequent rounds | - | - | 92% | 93% | 91% | - | - |  |
| Liberia | First available round | 97% | 75% | 51% | 15% | 48% | 54% | 94% | **0.8** |
|  | Average over subsequent rounds | 69% | 86% | 39% | 22% | 51% | 65% | - |  |
| Malawi | First available round | 78% | 97% | 67% | 48% | 81% | 77% | 82% |  |
|  | Average over subsequent rounds | - | 94% | - | - | - | 48% | - |  |
| Nigeria | First available round | 72% | 86% | 52% | 44% | 35% | 29% | 49% |  |
|  | Average over subsequent rounds | 66% | - | - | - | - | 26% | - |  |

**Note:** The spearman rank coefficient corresponds to the correlation between (i) the ranking of the resilience scores calculated using the first available round of survey data and the (ii) ranking of the resilience scores calculated using all subsequent rounds of survey data in each country. To obtain meaningful correlations, ranks are compared for countries which have at least 4 resilience domains with multiple rounds of survey data available. This excludes Chad (only 1 round of survey conducted), Guatemala and Malawi (only 2 domains with data across multiple rounds), Guinea and Nigeria (only 3 domains with data across multiple rounds). The rank correlation coefficient is bounded between -1 and +1 and is generally considered moderate when above or equal to 0.6 and strong when above or equal to 0.8. The “surge capacity” resilience domain is excluded from the table given that data were not available across multiple survey rounds.
